# Lack of Activity of Docetaxel in Soft Tissue Sarcomas: Results of a Phase II Study of the Italian Group on Rare Tumors

**DOI:** 10.1080/13577149977613

**Published:** 1999-12

**Authors:** Armando Santoro, Antonella Romanini, Alberto Rosso, Sergio Frustaci, Alessandro Comandone, Gaetano Apice, Domenico De Toma, Luigi Dogliotti, Rita Lionetto, Carla Dani, Paolo Bruzzi, Marco Piolini, Paola Bergnolo, Claudio Verusio

**Affiliations:** 1 Italian Group on Rare Tumors c/o Istituto Clinico Humanitas Via Manzoni, 56 Milano Rozzano 20089 Italy; 2 Ospedale Santa Chiara Pisa Italy; 3 Istituto San Raffaele Milano Italy; 4 Centro Riferimento Oncologico Aviano Italy; 5 Ospedale Gradenigo Torino Italy; 6 Fondazione Pascale Napoli Italy; 7 Ospedale San Donato Milano Italy; 8 Ospedale San Luigi Orbassano-Torino Italy; 9 IST Genova Italy; 10 Rhone Poulenc Rorer Italy

## Abstract

*Purpose.* The prognosis of advanced soft tissue sarcoma is poor, only a few drugs showing some activity with response rates around 15– 25%. Consequently drug development seems mandatory to improve treatment outcome. Following previous favourable EORTC experience, the Italian Group on Rare Tumors started a phase II study with docetaxel to confirm the activity of this drug in soft tissue sarcoma.

*Patients and methods.* Thirty-seven patients with soft tissue sarcoma resistant to at least one anthracyclinecontaining regimen were enrolled in a phase II multicenter study evaluating docetaxel 100 mg/m^2^ in a 1-h i.v. infusion q^3^ weeks.

*Results.*Thirty-seven patients were enrolled onto this phase II study and 36 were evaluable for response. Only one partial remission was observed [2.8% with 95% confidence interval (CI) 0.1– 16.2%]. Median progression-free and overall survival were 42 and 350 days, respectively. Neutropenia and leukopenia as well as cutaneous manifestations were the most common toxicities.

*Discussion.* The results of this phase II study do not confirm a previous EORTC repor t on the activity of docetaxel in soft tissue sarcoma, but are consistent with other more recent phase II studies. The accumulated evidence does not justify the use of this drug in the management of patients suffering from this disease, resistant to anthracyclinecontaining regimens.


Sarcoma (1999) 3, 177–181

ORIGINAL ARTICLE

### Lack of activity of docetaxel in soft tissue sarcomas: results of a phase II study of the Italian Group on Rare Tumors

ARMANDO SANTORO,[1] ANTONELLA ROMANINI,[2] ALBERTO ROSSO,[3] SERGIO FRUSTACI,[4] ALESSANDRO COMANDONE,[5] GAETANO APICE,[6] DOMENICO DE TOMA,[7] LUIGI DOGLIOTTI,[8] RITA LIONETTO,[9] CARLA DANI,[9] PAOLO BRUZZI,[9] MARCO PIOLINI,[10] PAOLA BERGNOLO[5] & CLAUDIO VERUSIO,[3] FOR THE ITALIAN GROUP ON RARE TUMORS

*[1]Istituto Clinico Humanitas, Rozzano-Milano, [2]Ospedale Santa Chiara, Pisa, [3]Istituto San Raffaele, Milano, [4]Centro Riferimento Oncologico, Aviano, [5]Ospedale Gradenigo, Torino, [6]Fondazione Pascale, Napoli, [7]Ospedale San Donato, Milano, [8]Ospedale San Luigi, Orbassano-Torino, [9]IST, Genova, [10]Rhone Poulenc Rorer*

**Abstract**

*Purpose.* The prognosis of advanced soft tissue sarcoma is poor, only a few drugs showing some activity with response rates around 15–25%. Consequently drug development seems mandatory to improve treatment outcome. Following previous favourable EORTC experience, the Italian Group on Rare Tumors started a phase II study with docetaxel to confirm the activity of this drug in soft tissue sarcoma.

*Patients and methods.* Thirty-seven patients with soft tissue sarcoma resistant to at least one anthracycline-containing regimen were enrolled in a phase II multicenter study evaluating docetaxel 100 mg/m$^2$ in a 1-h i.v. infusion q3 weeks.

*Results.* Thirty-seven patients were enrolled onto this phase II study and 36 were evaluable for response. Only one partial remission was observed [2.8% with 95% confidence interval (CI) 0.1–16.2%]. Median progression-free and overall survival were 42 and 350 days, respectively. Neutropenia and leukopenia as well as cutaneous manifestations were the most common toxicities.

*Discussion.* The results of this phase II study do not confirm a previous EORTC report on the activity of docetaxel in soft tissue sarcoma, but are consistent with other more recent phase II studies. The accumulated evidence does not justify the use of this drug in the management of patients suffering from this disease, resistant to anthracycline-containing regimens.

**Key words:** *docetaxel, soft tissue sarcoma*

### Introduction

The prognosis of advanced soft tissue sarcoma is poor, with only three drugs, namely anthracyclines, ifosfamide and dacarbazine, showing some activity with a response rate of around 15–25%.[1–4] The combination of such drugs does not significantly increase the response rate over single-drug doxorubicin.[5–9] Consequently new drug development seems mandatory to improve the treatment outcome of patients suffering from this disease. Unfortunately almost all phase II studies of new agents performed in recent years failed to show any activity in the treatment of refractory soft tissue sarcomas.[5,9,10]

Docetaxel (Taxotere®) is a semisynthetic analog of paclitaxel derived from the needles of the European yew, *Taxus baccata*. Docetaxel was shown to have superior *in vivo* antitumor activity as compared to taxol in the B16 melanoma model[11] and has shown a favorable response rate in breast cancer.[12]

A phase II study of docetaxel by the EORTC Soft Tissue and Bone Sarcoma Group[13] reported an overall response rate of 17% in 29 soft tissue sarcoma patients refractory to an anthracycline-containing regimen. According to these data, docetaxel has been suggested as the third active agent in the treatment of advanced soft tissue sacoma supporting its use as a first-line treatment.

In September 1995, the Italian Group on Rate Tumors started a multicenter phase II study in refractory soft tissue sarcoma to confirm previous EORTC experience.

Correspondence to: A. Santoro, MD, Italian Group on Rare Tumors, c/o Istituto Clinico Humanitas, Via Manzoni, 56, 20089 Rozzano, Milano, Italy. Tel. +39 2 8224–4541; Fax. +39 2 8224–4591; e-mail: armando.santoro@humanitas.it

1357-714X print/1369-1643 online/99/040177-05 © 1999 Taylor & Francis Ltd

### Patients and methods

### *Patient population*

To be eligible for the study, patients were required to have a pathologically confirmed diagnosis of soft tissue sarcoma and to be resistant to at least one anthracycline-containing regimen.

Resistance to previous chemotherapy was defined as either progression during or relapse within 6 months from the end of previous chemotherapy. Eligible patients were to have received no regimens including docetaxel. Other inclusion criteria were as follows: age between 16 and 65 years, ECOG performance status ≤2, an expected survival duration of ≥ 3 months, and previous chemotherapy completed at least 4 weeks before study entry. Patients were required to have bidimensionally measurable indicator lesions, while patients with evaluable lesions alone as well as those with pleural effusion, malignant ascites and previously irradiated lesions were not considered for enrollment in the study. Requirements for eligibility also included leukocyte count ≥3500 cmm; granulocyte count ≥1500 cmm; platelet count ≥100 000 cmm, and normal liver and renal function. Patients with cardiac disease, brain metastases, other serious medical illnesses and other malignant tumors were excluded. Protocol and information sheet were approved by the Scientific and Ethical Committee of all the participating institutions and all patients had to sign a written, informed consent.

### *Treatment plan*

Before treatment, all patients had a complete history and physical examination as well as baseline laboratory tests, chest X-rays, ECG, and abdominal ultrasound. CT scan, MRI and bone scan were performed according to the clinical indications.

Docetaxel was supplied by Rhone Poulenc Rorer company as a concentrated sterile solution with 40 mg/ml (80 mg/2 ml, per vial) in polysorbate 80 (Tween 80). Before infusion, docetaxel was diluted in 250 ml of 5% dextrose and was given at a dose of 100 mg/m$^2$ i.v., over 1 h. Cycles were restarted every 21 days in the absence of toxicities requiring treatment delay. Therapy was continued until disease progression or unacceptable toxicity.

The patients did not receive anti-emetic treatment unless the patient had experienced nausea and vomiting with a previous dose. Pre-medication for acute hypersensitivity reactions consisted of prednisone 50 mg orally 24, 16, 8 and 1 h before and 20, 32, 44, 56, 68 and 80 h after docetaxel. Prophylactic colony-stimulating factors were not given. Toxic effects were reported according to the South West Oncology Group Toxicity Criteria.[14] Dose reductions were performed as follows: in patients with grade 3 neutropenia and/or grade 2 peripheral neuropathy and/or febrile neutropenia the following cycle was lowered of a 25%; in the absence of recovery of myelotoxicity after 3 weeks, the subsequent dose was delayed until toxicity resolved.

### *Response criteria*

Response evaluation was performed every two cycles reporting the initial parameter lesions. According to the South West Oncology Group criteria,[14] complete remission was defined as the complete disappearance of all detectable lesions for at least 4 weeks. Partial remission was defined as a ≥50% decrease in the sum of the products of the diameters of measurable lesions for at least 4 weeks, in the absence of simultaneous increase of any lesion and/or appearance of any new lesion. Progressive disease was defined as an increase of ≥50% in the size of a measurable lesion or the appearance of a new lesion. Duration of response was determined by the interval between the day of the first treatment and the date of clear evidence of disease progression.

### *Statistical analysis*

The study was designed as a two-stage trial, according to Simon's optimal design,[15] with the following specifications: a 90% probability of accepting docetaxel for further studies was required if the true response rate was 20% whereas the probability of accepting it for further studies, if the true response rate was 10%, was set at 5%. Accordingly, 12 patients had to be entered and evaluated in the first stage of the trial. If at least one response was observed, 25 more patients had to be accrued, and, overall, at least four responses had to be observed out of 37 patients in order to consider the drug sufficiently active to warrant further studies.

All patients who started the experimental treatment were included in the analyses of response. All patients in whom an objective response was not demonstrated, including early progressions and deaths, those who discontinued treatment due to toxicity, and those in whom response was not evaluated according to the protocol were considered as treatment failures and included in the denominator of the proportion of responses.

Survival and time to disease progression were evaluated according to the Kaplan–Meier method.[16]

### Results

### *Patient characteristics*

Between September 1995 and October 1996, 37 patients were enrolled onto this phase II study by nine participating centres. Out of these 37 patients, five were not eligible because of performance status 3 (one case), low neutrophiles <2000 cmm (two cases) and abnormal renal function (two cases), respectively. All patients had measurable lesions. Pre-treatment patient characteristics are listed in Table 1. The median age was 44 years (range 20–64 years) with a median WHO performance status of 0 (range 0–3). Thirty-four patients (91.9%) presented with metastatic disease and only three (8.1%) with locoregional advanced disease. Twenty-four patients (64.9%) were pre-treated with only one chemotherapy regimen, nine (24.3%) with two, and four with four, respectively. Previous radiotherapy had been administered to 19 patients. The histologic subtypes were as follows: leiomyosarcoma in nine cases, synovial sarcoma in eight cases, malignant fibrous histiocytoma and schwannoma in four cases, fibrosarcoma, liposarcoma and rhabdomyosarcoma in two cases, emangiopericytoma and epithelioid sarcoma in one case, and unclassified sarcoma in four cases, respectively.

### *Response*

All 37 patients were considered evaluable for response except one who died before starting chemotherapy. A total of 116 cycles were administered with a median of four courses (range 1–8). The therapeutic results are reported in Table 2. Only one partial response (2.8% with a 95% confidence interval 0.1–16.2%) was observed. In 10 patients, stable disease was documented, while all other patients showed disease progression.

The median time to progression was 42 days, and median overall survival was 350 days, respectively (Table 2 and Fig. 1). Death was due to tumor progression in 31 patients, to infection in one patient not eligible because of both anemia and abnormal renal function, and cause of death was not documented in two cases.

### *Toxicity*

The highest grade of toxicity according to the SWOG Toxicity Criteria[14] for each patient is listed in Table 3. Grade 3 and 4 neutropenia and leukopenia were the most common toxicities, while anemia and thrombocytopenia were rarely documented. Febrile neutropenia and infection were observed in a small proportion of cases although only one patient died of this cause. More than half of the patients developed symptomatic rash as well as more severe cutaneous manifestations including superficial dry desquamation of the hands and feet and dystrophic nail changes. Hair loss was observed in almost all evaluable patients. Vomiting and diarrhea, mucositis and neurotoxicity were rarely reported. Fluid retention occurred in 21% of patients but only in 6% of cases reached grade 2–3 level and a hypersensitivity reaction was observed in 9% of cases.

**Table 1.** *Pretreatment patient characteristics*

| No. of patients entered | 37 |
|---|---|
| Median age in years (range) | 44 (20–64) |
| Sex, Males/Females | 19/18 |
| WHO performance status | |
| Median (range) | 0 (0–3) |
| Site of disease | |
| Metastatic | 33 (89.2%) |
| Locoregional | 3 (8.1%) |
| Both | 1 (2.7%) |
| No of previous chemotherapy regimens | |
| 1 | 24 (64.9%) |
| 2 | 9 (24.3%) |
| >2 | 4 (10.8%) |
| Previous radiotherapy | 19 (51.4%) |

**Table 2.** *Response evaluation*

| | No | |
|---|---|---|
| Patients entered | 37 | |
| Patients evaluated | 36 | |
| Response | | |
| Complete | 0 | |
| Partial | 1 | 2.8% |
| Stable | 10 | |
| Failure | 25 | |
| Median duration in days | | |
| Progression-free survival | 42 | |
| Overall survival | 350 | |
| Deaths | 36 | |

*95% confidence interval 0.1–16.2%

### Discussion

Both doxorubicin and ifosfamide have been considered as the most active drugs in the management of adult soft tissue sarcoma.[1–3,5,9] Some reports have also suggested a low level of activity of dacarbazine.[4,9] Nonetheless the results achieved with these drugs given alone or in combination are poor with no clear benefit in the advanced disease as well as in the adjuvant setting.[1,5,9] Therefore the search for new active agents remains an important issue. However the majority of phase II studies failed to show any activity by almost all investigated drugs.[1,5,9,10]

The EORTC Soft Tissue and Bone Sarcoma Group reported the results of docetaxel in previously treated soft tissue sarcoma with five partial remissions (17%) in 29 evaluable patients.[13] Based on these positive results it was suggested that docetaxel could be an effective drug, warranting first-line phase II studies.

However, the results obtained in the present study failed to confirm the activity of this drug in soft tissue sarcoma with only one partial remission in 37 anthracycline-resistant patients. Our results are in agreement with the low response rate reported by three studies utilizing docetaxel as first-line.[17–19] Based on our data and that of other authors, the continued use of docetaxel for treatment of soft tissue sarcoma is not justified.

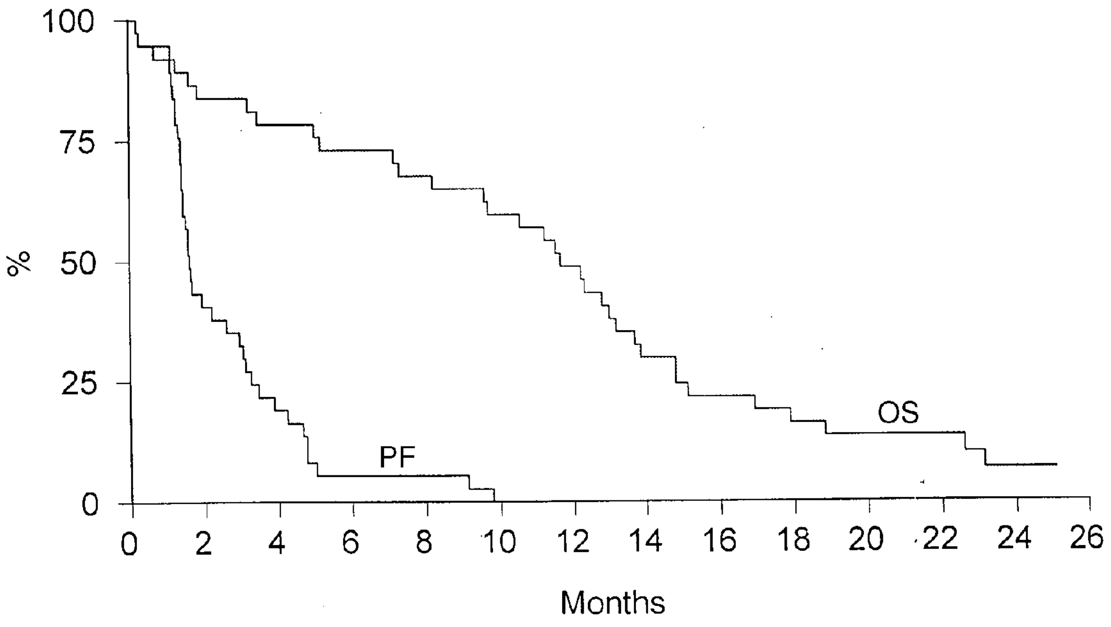

**Figure 1.** *Progression-free (PF) and overall survival (OS) of patients with refractory soft tissue sarcoma after treatment with docetaxel.*

**Table 3.** *Toxicity of docetaxel*

| Adverse effects | Highest SWOG Toxicity Grade* | | | | |
|---|---|---|---|---|---|
| | 0 | 1 | 2 | 3 | 4 |
| Neutropenia | 6 | — | 3 | 15 | 76 |
| Leukopenia | 3 | 6 | 15 | 44 | 32 |
| Anemia | | 74** | 17 | 9 | 0 |
| Thrombocytopenia | 85 | 12 | 0 | 0 | 3 |
| Infection | 70 | 6 | 9 | 12 | 3 |
| Rash | 82 | 15 | 3 | 0 | 0 |
| Cutaneous | 59 | 23 | 15 | 3 | 0 |
| Vomiting | 85 | 3 | 9 | 3 | 0 |
| Fever | 76 | 9 | 12 | 0 | 3 |
| Neurotoxicity | 68 | 29 | 3 | 0 | 0 |
| Fluid retention | 79 | 15 | 3 | 3 | 0 |
| Hypersensitivity | 91 | 3 | 0 | 3 | 3 |
| Mucositis | 62 | 23 | 6 | 9 | 0 |
| Diarrhea | 68 | 17 | 9 | 6 | 0 |

*% of patients.
** Grade 0–1.

### References

1 Santoro A, Soto Parra H. Sarcoma. In: *Textbook of Medical Oncology* 1999; in press.

2 Mouridsen HT, Bastholt L, Somers R, *et al.* Adriamycin versus epirubicin in advanced soft tissue sarcomas. A randomized phase II/phase III study of the EORTC Soft Tissue and Bone Sarcoma Group. *Eur J Cancer Clin Oncol* 1987;23:1477–83.

3 Bramwell VH, Mouridsen H, Santoro A, *et al.* Cyclophosphamide versus ifosfamide: final results of a randomized phase II trial in adult soft tissue sarcoma. *Eur J Cancer Clin Oncol* 1987;23:311–21.

4 Buesa JM, Mouridsen HT, van Oosterom AT, *et al.* High-dose DTIC in advanced soft-tissue sarcomas in the adult. A phase II study of the EORTC Soft Tissue and Bone Sarcoma Group. *Ann Oncol* 1991;2:307–9.

5 Santoro A, Soto Parra H. Soft tissue and bone sarcomas. *Cancer Chemotherapy and Biological Response Modifiers Annual* 1998;18 (in press).

6 Santoro A, Tursz T, Mouridsen H, *et al.* Doxorubicin versus CYVADIC versus doxorubicin plus ifosfamide in first-line treatment of advanced soft tissue sarcomas: a randomized study of the European Organization for Research and Treatment of Cancer, Soft Tissue and Bone Sarcoma Group. *J Clin Oncol* 1995;13:1537–45.

7 Antman K, Crowley J, Balcerzak SP, *et al.* An intergroup phase III randomized study of doxorubicin and dacarbazine with or without ifosfamide and mesne in advanced soft tissue and bone sarcomas. *J Clin Oncol* 1993;11:1276–85.

8 Edmonson JH, Ryan LM, Blum RH, *et al.* Randomized comparison of doxorubicin alone versus ifosfamide plus doxorubicin or mitomycin, doxorubicin and cisplatin against advanced soft tissue sarcomas. *J Clin Oncol* 1993;11:1269–75.

9 Santoro A. Advanced soft tissue sarcoma: How many more trials with anthracyclines and ifosfamide? *Ann Oncol* 1999;10.

10 Blackledge G, van Oosterom AT, Mouridsen HT, *et al.* Doxorubicin in relapsed soft tissue sarcoma: Justification of phase II evaluation of new drugs in this disease. *Eur J Cancer Clin Oncol* 1990;26:134–41.

11 Extra JM, Rousseau F, Bruno R, Clavel M, Le Bail N, Marty M. Phase I and pharmacokinetic study of Docetaxel (RP 56976; NSC 628503) given as a short intravenous infusion. *Cancer Res* 1993;53(5):1037–42.

12 Hortobagyi G. An expanding role for docetaxel. *Semin Oncol* 1998;25:(6 Suppl 13):1–3.

13 Van Hoesel QGCM, Verweij J, Catimel G, *et al.* Phase II study with Docetaxel (Taxotere ®) in advanced soft tissue sarcomas of the adult. *Ann Oncol* 1994;5:539–42.

14 Green S, Weiss GR. Southwest Oncology Group standard response criteria, endpoint definitions and toxicity criteria. *Invest New Drugs* 1992;10:239–53.

15 Simon R. Optimal two-stage designs for phase II clinical trials. *Controlled Clinical Trial*, 1989;10:1–10.

16 Kaplan EL, Meier P. Nonparametric estimation from incomplete observations. *J Am Statist Assoc* 1958;53:457–81.

17 Edmonson JH, Ebbert LP, Nascimento AG, *et al.* Phase II study of docetaxel in advanced soft tissue sarcomas. *Am J Clin Oncol* (CCT) 1996;19:574–6

18 Verweij J, Judson I, Crowther D, *et al.* Randomized study comparing docetaxel (Taxotere® (T) to doxorubicin (D) in previously untreated soft tissue sarcomas (STS). *Proc. Am Soc Clin Oncol* 1997;16:496a.

19 Bramwell V, Blackstein M, Belanger K, *et al.* A phase II study of docetaxel in chemotherapy-naive patients with recurrent or metastatic adult soft tissue sarcoma. *Sarcoma* 1998;2:29–33.
